# The trend of 10-year cardiovascular risk among diabetic and non-diabetic participants in Tehran Lipid and glucose study: 1999–2018

**DOI:** 10.1186/s12889-022-12981-9

**Published:** 2022-03-28

**Authors:** Fatemeh Koohi, Karim Kohansal, Marzieh Saei Ghare Naz, Somayeh Derakhshan, Fereidoun Azizi, Davood Khalili

**Affiliations:** 1 Obesity Research Center, Research Institute for Endocrine Sciences, Shahid Beheshti University of Medical Sciences, Tehran, Iran; 2 Prevention of Metabolic Disorders Research Center, Research Institute for Endocrine Sciences, Shahid Beheshti University of Medical Sciences, Tehran, Iran; 3 Reproductive Endocrinology Research Center, Research Institute for Endocrine Sciences, Shahid Beheshti University of Medical Sciences, Tehran, Iran; 4 Department of Epidemiology, School of Public Health and Safety, Shahid Beheshti University of Medical Sciences, Tehran, Iran; 5 Endocrine Research Center, Research Institute for Endocrine Sciences, Shahid Beheshti University of Medical Sciences, Tehran, Iran

**Keywords:** Cardiovascular risk score, Risk factors, Diabetes, Trend, Cohort study

## Abstract

**Background:**

Assessing the risk of cardiovascular disease (CVD) is crucial in preventive cardiology. We aimed to determine the trend of CVD risk among individuals with and without diabetes during two decades of follow-up in a Middle Eastern cohort.

**Methods:**

We studied 8,450 individuals (55.5% women) aged 40–75 years who participated in the Tehran Lipid and Glucose Study (TLGS). Diabetes status and CVD risk factors were evaluated in six examinations from 1999 to 2018. The individual 10-year CVD risk score was calculated using the ACC/AHA recommended risk equation. We used generalized estimating equation models (GEE) to assess the time trends of CVD risk factors and CVD risk scores in diabetic and non-diabetic groups separately.

**Results:**

The age-adjusted ACC/AHA risk score significantly decreased in non-diabetic women and men (from 3.2% to 1.6% in women and 6.8% to 5.0% in men; *p* for trend < 0.001). Whereas the risk significantly decreased among diabetics men (from 13.8% to 11.5%), it increased somehow among diabetics women (from 5.3% to 5.5%). Furthermore, in both sexes, diabetic individuals compared to non-diabetic ones had better control on their systolic blood pressure, total cholesterol, and fasting plasma glucose during the last two decades.

**Conclusions:**

The CVD risk and most CVD risk factors improved in individuals with and without diabetes in the past two decades; however, they have not reached the targets yet. So, more stringent lifestyle modifications and treatment strategies are needed, especially for primary prevention in the general population.

**Supplementary Information:**

The online version contains supplementary material available at 10.1186/s12889-022-12981-9.

## Introduction

Cardiovascular disease (CVDs) is one of the leading causes of death worldwide [1]. More than 80 percent of the global burden of CVD is in low- and middle-income countries [2]. Death rates from CVD are expected to rise to 23.6 million by 2030 [3]. CVD prevalence varies in different countries depending on their health care system, culture, and economy [4]. In Iran, CVD is also among the leading and primary causes of death [5], with 43% deaths in 2016 [6]. Because of population aging over the past decades, the burden of CVD has increased in Iran. It is projected that the years lost due to disability will become more than double from 2005 to 2025 [7].

In the last two decades, the geographical distribution of CVD has changed dramatically with a decline in developed countries and an increase in developing countries, including the Middle East. In Iran also, western lifestyles such as eating habits, smoking, and physical inactivity resulted in increasing CVD risk factors during the last decades. This situation persuaded the policymakers for improving lifestyle modification and preventive strategies in the country [8, 9].

CVD risk screening is a well-known primary prevention strategy by calculating a risk score that combines known cardiovascular risk factors [10]. This risk assessment is used extensively to identify high-risk individuals and perform appropriate interventions [11]. One of the risk assessment tools is the pooled equation recommended by the American College of Cardiology (ACC) and the American Heart Association (AHA) that assess the probability of CVD events over ten years. The ACC/AHA risk model uses major cardiovascular risk factors, including age, current smoking, diabetes, systolic blood pressure, total cholesterol, and HDL cholesterol, to assess cardiovascular risk [12].

Given the importance of risk of CVD, this study aimed to determine the trend of cardiovascular risk based on the ACC/AHA risk model and also the trend of its components among individuals with and without diabetes during two decades follow-up in a Middle Eastern cohort.

## Material and methods

### Study population

This study used data from the Tehran Lipid and Glucose Study (TLGS), a population-based cohort study conducted in 1999 to assess the risk factors of non-communicable diseases, including cardiovascular events. A detailed description of TLGS is available elsewhere [13, 14]. Briefly, a total of 15,005 and an additional 3555 individuals aged three years and over who were residents of district No.13 of Tehran were selected in exam 1 (1999 to 2001) and exam 2 (2002–2005), respectively. The participants repeatedly have been followed up in four other subsequent examination cycles with approximately 3-year intervals: exam 3 (2006 to 2008), exam 4 (2009 to 2011), exam 5 (2012 to 2014), and exam 6 (2015 to 2017).

**Fig. 1:**
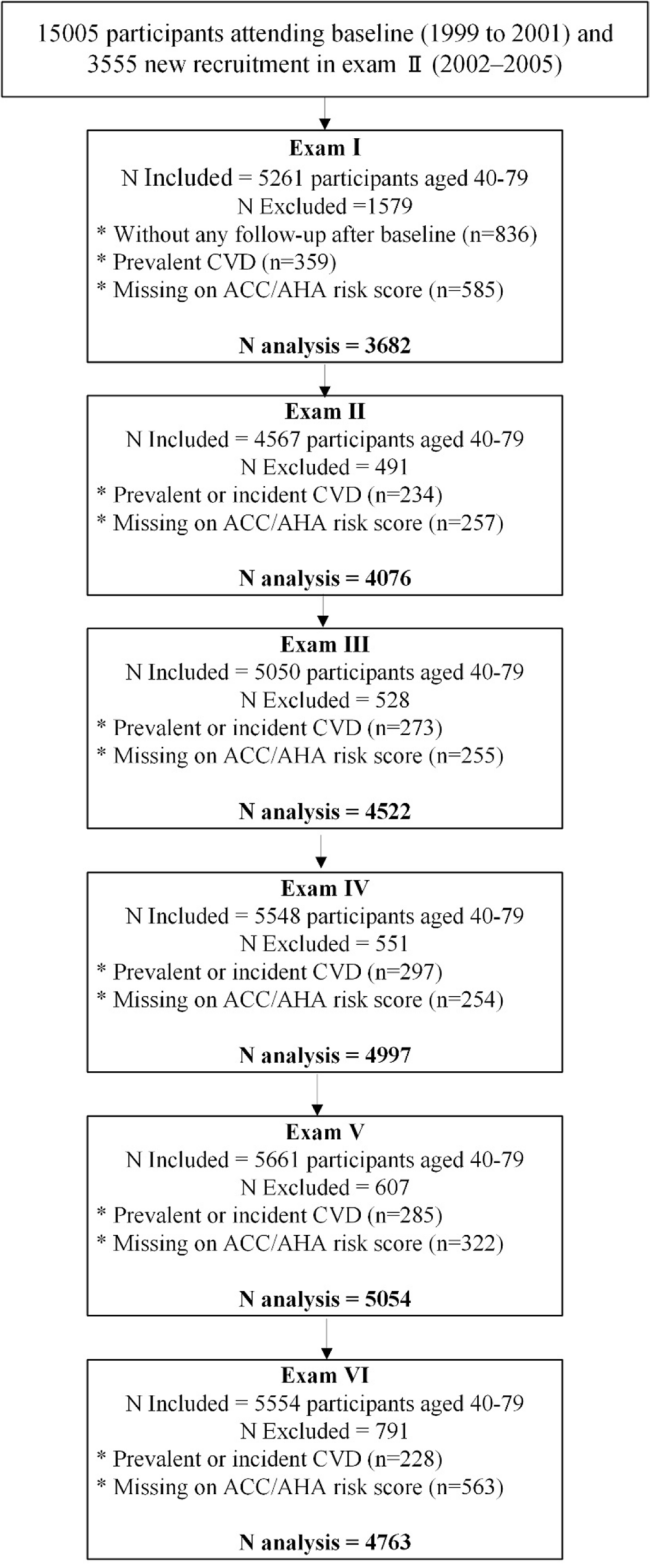
Flowchart of the participants included at each examination cycle

According to the required criteria for the ACC/AHA risk assessment model, individuals aged 40–79 years and were free of cardiovascular disease in each examination cycle were considered for the current study. We excluded participants with no follow-up after the baseline assessment (*n* = 836) and those with a history of CVD and missing on variables needed for ACC/AHA risk assessment in each exam. Thus, the final sample included 8450 participants (2064 diabetic and 6386 non-diabetic subjects) with a median period of 10 years (Inter-quartile range: 8 years) and 27,094 person-exam. A flowchart of the participants included in each examination cycle is shown in Fig. 1.

### Measurements and definitions

The Pooled Risk Equations recommended by ACC/AHA for non-Hispanic white men and women were used to calculate the 10-year risk of hard CVD [5]. The risk score components were drawn from standard questionnaires, physical exams, and laboratory measurements [13]. The covariates included in this equation are as follow: systolic blood pressure, taking hypertension medication, serum total cholesterol, diabetes defined as fasting plasma glucose ≥7.0 mmol/l (126 mg/dl) or taking any medication for diabetes, HDL-cholesterol and current smoking status defined as using any tobacco product (cigarette, pipe, water pipe) regularly or occasionally.

### Statistical methods

Demographic data and other clinical information related to study participants were presented as mean and standard deviation or frequency and percent. Cross-sectional data were linked across the study exams to perform trend-analysis of ACC/AHA risk score, systolic blood pressure (SBP), total cholesterol (TC), HDL-C, fasting plasma glucose (FPG), and current smoking. The GEE analysis, with autoregressive working correlation structures, through identity link function with Gaussian family, was used to consider the correlation between the repetitions of some individuals in different exams.

The marginal (age-adjusted) means in each exam and *P* values for the time trend were computed according to the GEE models fitted separately for diabetic and non-diabetic groups. The interaction between the diabetes status and each exam of the study was checked by entering the cross-product term (interaction term) between diabetes status and time in a separate model, including diabetic and non-diabetic subjects together. Since age is a strong component of CVD risk score and increases during follow-up, we adjusted all statistical models for the participants' age in each exam to eradicate the potential confounding effect of age. All statistical analyses were performed separately in men and women using STATA version 14 statistical software.

## Results

### Populations characteristics

Out of the 8450 participants in this study, 4687 (55.5%) were women, and 3763 (44.5%) were men. Diabetic participants in all examination cycles were older. The age and sex distribution of diabetic and non-diabetic participants across the six examination cycles are shown in Table 1.

**Table 1:** Age and sex distribution of the participants in each exam by diabetes status

Characteristic	Examination cycles					
	**I**	**II**	**III**	**IV**	**V**	**VI**
**Total**						
No. of participants	3682	4076	4522	4997	5054	4763
Age (y), mean ± SD	53.0 ± 9.2	53.7 ± 10.0	53.6 ± 10.1	53.7 ± 10.2	54.1 ± 10.2	54.7 ± 9.9
Sex, Women (%)	2094 (56.9)	2333 (57.2)	2591 (57.3)	2826 (56.6)	2859 (56.6)	2748 (57.7)
**Diabetics**						
No. of participants (%)	658 (17.9)	732 (18.0)	723 (16.0)	926 (18.5)	986 (19.5)	992 (20.8)
Age (y), mean ± SD	55.8 ± 8.9	57.3 ± 9.5	57.6 ± 9.7	58.3 ± 9.8	59.0 ± 10.2	59.7 ± 9.6
Sex, Women (%)	411(62.5)	450(61.5)	441(61.0)	564(60.9)	582(59.0)	594(59.9)
**Non-Diabetics**						
No. of participants (%)	3024 (82.1)	3344 (82.0)	3799 (84.0)	4071 (81.5)	4068 (80.5)	3771 (79.2)
Age (y), mean ± SD	52.4 ± 9.2	52.9 ± 9.9	52.8 ± 10.0	52.7 ± 10.0	52.9 ±	53.4 ± 9.5
Sex, Women (%)	1683(55.7)	1883(56.3)	2150(56.6)	2262(55.6)	2277(56.0)	2154(57.1)

**Fig. 2:**
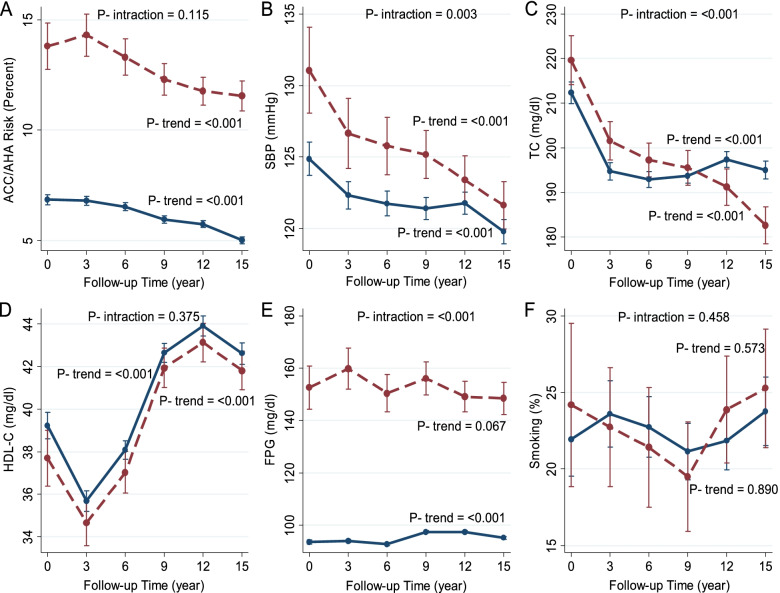
Age-adjusted marginal means of ACC/AHA CVD risk score (**A**), systolic blood pressure (**B**), total cholesterol (**C**), HDL- cholesterol (**D**), fasting plasma glucose (**E**), and current smoking (**F**) in each phase in men separated by diabetes status. Dash lines show the diabetic group, and solid lines show the non-diabetic group. Models for assessment of time trend were fitted separately for diabetic and non-diabetic subjects, and age-adjusted marginal means and *p*-values for trend were reported. Meanwhile, the interaction of diabetes status with time was assessed by fitting a model including diabetes status, the follow-up time and their cross-product in a pooled model including both diabetic and non-diabetic participants, and *p*-values for the cross-product term were reported as p for trend

**Fig. 3:**
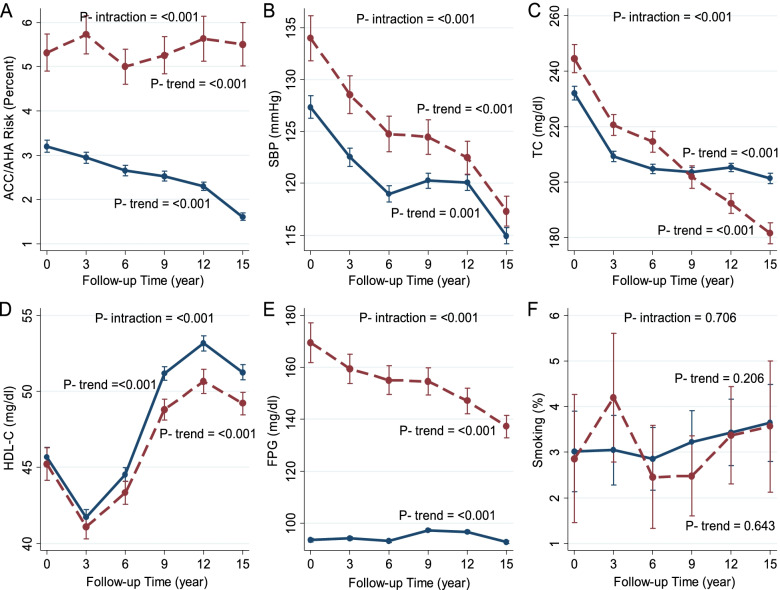
Age-adjusted means of ACC/AHA CVD risk score (**A**), systolic blood pressure (**B**), total cholesterol (**C**), HDL-cholesterol (**D**), fasting plasma glucose (**E**), and current smoking (**F**) in each phase in women separated by diabetes status. Dash lines show the diabetic group, and solid lines show the non-diabetic group. Models for assessment of time trend were fitted separately for diabetic and non-diabetic subjects, and age-adjusted marginal means and *p*-values for trend were reported. Meanwhile, the interaction of diabetes status with time was assessed by fitting a model including diabetes status, the follow-up time and their cross-product in a pooled model including both diabetic and non-diabetic participants, and *p*-values for the cross-product term were reported as p for trend

Figures 2 and 3 demonstrate the age-adjusted marginal means of ACC/AHA risk score and CVD risk factors in each exam separated by diabetes status in men and women, respectively. The values of these age-adjusted marginal means are also shown in Supplementary Table S1.

### Changes in the ACC/AHA CVD risk score

We observed a significant decrease in the mean 10-year age-adjusted ACC/AHA risk score in non-diabetic women and men (from 3.2% to 1.6% in women and 6.8 to 5.0% in men; *p* for trend < 0.001). In diabetic men, the risk decreased from 14.3% in exam II to 11.5% in exam VI (*p* for trend < 0.001). Also, among women with diabetes, there was a slight rising trend for ACC/AHA risk score in two-point of study (exam I-II: from 5.3% to 5.7% and exam III-V: from 5.0% to 5.6%; *p* for trend < 0.001). For men, the *P*-value for interactions between time and diabetes status was 0.115; however, the interaction was statically significant in women (*p* for interaction < 0.001).

### Changes in the mean of CVD risk factors

The trend analysis showed significant reductions of the age-adjusted mean of SBP among diabetic participants (from 133.3 to 117.6 mmHg in women and 131.0 to 121.6 mmHg in men; for trend < 0.001). Besides, among non-diabetic men and women, the trend was generally decreasing with some fluctuations. (For both men and women, *P*-value for interactions between the time and diabetes status were significant < 0.05).

Our results showed significant reductions of age-adjusted mean of TC among diabetic participants (from 241.9 to 184 mg/dl in women and 219.6 to 182.6 mg/dl in men; for trend < 0.001). Also, the result showed a reduction trend of the age-adjusted mean of TC from exam I to exam III and after that with some fluctuations in non-diabetic men and women. (*p* for trend < 0.0001).

Among all diabetic and non-diabetic participants, there was a similar sharp decrease of the age-adjusted mean of HDL from exam I to exam II. Following this trend, a similar sharp increase from exam II to exam IV was observed. Again, a decreasing trend was repeated between exam V to exam VI. (*p* for trend was < 0.0001 generally). (*P*-value for interactions between the follow-up time and diabetes status were 0.375 and < 0.001, for men and women, respectively).

There were non-significant decreasing trends in age-adjusted mean of FPG from exam II to III and between exams IV to VI among diabetic men. (*p* for trend = 0.067). In the diabetic state, women experienced a significant decreasing trend in the age-adjusted mean of FPG (from 164.2 to 134.2; *p* for trend < 0.0001). In the non-diabetic state, there was a similar flat trend from exam I to exam III in both men and women. (For both men and women, *P*-value for follow-up time and diabetes status interactions were < 0.001).

There were no significant trends in age-adjusted current smoking among diabetic and non-diabetic participants for both sexes (p for trend > 0.05). (For both men and women, *P*-value for interactions between follow-up time and diabetes status were 0.456 and 0.706, respectively).

## Discussion

This study assessed the time trends of ACC/AHA risk score and risk factors among TLGS diabetic and non-diabetic participants over two decades of follow-up. Researches on CVD risk scores are limited, and here, we have assessed CVD risk trends in our population to provide a better perspective on future prevention and treatment strategies. The time trend analyses in our study indicated that the ACC/AHA risk score decreased over time in both diabetic and non-diabetic men. However, the direction of movement among women with diabetes was not the same; diabetic women's CVD risk scores have increased somehow during follow-up. We also observed significant improvements in systolic blood pressure, total cholesterol, HDL cholesterol, and fasting blood glucose levels in diabetic and non-diabetic individuals, with more improvements in SBP, TC, and FPG in diabetic subjects than those without this condition.

In our study, non-diabetic subjects showed favorable trends in risk scores for both sexes, decreasing from 3.1% to 1.6% for women and 6.8% to 5.01% for men. In the diabetic population, although men's risk score dropped from 13.79 to 11.54, women's risk score fluctuated over time and generally did not show a favorable trend. Consistent with our findings, in a previous study among the French population, there were downward trends in the 10-year CVD risk using two scoring methods (Framingham and European System Coronary Artery Risk Assessment) [15]. In two studies on the US population to examine the trend of CVD risk, the overall trend was not significant [16, 17].

To better understand the trends and directions of the population's risk score, it is best to examine cardiovascular risk factors as components of the risk scores separately. In our study, risk factor levels were higher in people with diabetes than non-diabetics, which is in line with the previous studies showing a correlation between diabetes and other cardio-metabolic risk factors [9, 18, 19]. Moreover, a recent multicenter trial study has confirmed the close association between CVD events and the number of risk factors at target in the diabetic population [20]. This study has shown that a multifactorial intervention on multiple modifiable risk factors in diabetic kidney disease patients, including hyperglycemia, hypertension, and dyslipidemia, could reduce major fatal/non-fatal cardiovascular events in a few years and with long durability [20].

High blood pressure is a leading risk factor for almost all kinds of cardiovascular diseases [21] that showed a decreasing trend among both diabetic and non-diabetic participants in our study. These findings are consistent with other studies on the Iranian population, indicating that systolic blood pressure has been better controlled in the last decade, and the prevalence of high blood pressure has decreased. Nevertheless, these declines are not enough, and more strategies and plans in the health care system are needed to achieve the goals [22]. A systematic review reported that about a quarter of the Iranian population suffers from high blood pressure, increasing with aging, underscoring the burden of high blood pressure on our health care system [23]. A study on the US population showed similar decreasing trends in the prevalence of hypertension from 47.0 in 1999–2000 to 41.7 in 2013–2014 [24]. In contrast, Zheng et al.'s study among more than eighty thousand Chinese participants over the 24-year follow-up reported an increasing trend in the age-adjusted hypertension rate from 32.2% in 1991 to 60.0% in 2015 [25].

We revealed improvements in total cholesterol levels with a more significant beneficial reduction in people with diabetes than the non-diabetics; however, the cholesterol levels did not reach targets in both the diabetic and non-diabetic groups. Our findings are in line with those of previous studies, showing similar decreasing trends among Iranian populations [26, 27]. These results are also in line with those from the National Health And Nutrition Examination Survey (NHANES), which reported more improvements in TC levels in diabetic women than in non-diabetic women [28]. It seems that the increase in the use of antihypertensive agents and improving individuals' general knowledge are the crucial causes of these improvements [9, 27]. An 11-year follow-up study on the Korean population showed no favorable trend in TC in participants who did not use lipid-lowering drugs in both men and women [29]. Also, the decrease in the Iranian population's hydrogenated oils consumption might be another reason for these favorable trends in lipid profile, which has occurred in recent decades [30, 31].

HDL-C is another component of individuals' lipid profiles that showed arbitrary trends in our population. Different directions regarding HDL-C levels have been reported from other populations. In the Palmer and Toth study, the mean HDL-C levels of American men and women remained constant during the 10-year follow-up period [32]. Another survey of the Chinese population reported a growth in the HDL-C levels from 1998 to 2015 [16].

Globally, since 1980, the age-standardized mean FPG has increased by approximately 0.07 and 0.09 mmol/L per decade for men and women, respectively [33]. Our findings regarding the trend of FPG indicated poor glycemic control in diabetic men than in diabetic women. Besides, in diabetic people, FPG has changed favorably during the study period, but there was a slight rise in FPG levels in non-diabetic individuals. A study on the US population found that US diabetics who achieved glycemic control (HbA1c < 7.0%) increased from 49.6% in 1999–2004 to 58.6% in 2005–2010 [34]. Our results regarding differences in trends of FPG levels between diabetic and non-diabetic individuals are in line with those of a previous study on the diabetic and non-diabetic [18], indicating more success in secondary prevention strategies than in primary-level in the Iranian population. Further development in the diabetic community than in the non-diabetic community brings to mind that people are paying more attention to their lifestyle after developing diabetes and increasing awareness of the consequences of high blood glucose.

Contrary to the results of the previous studies, indicating a significant reduction in the prevalence of daily smoking in the Iranian population [18, 35], this study showed no significant change in the prevalence of smoking in participants with and without diabetes. Consistent with our study, Nemati et al. showed a steady trend of smoking among Iranian men and women from 2006 to 2009 [36]. The World Health Organization (WHO) reported a decline in the prevalence of tobacco use from 33.3% in 2000 to 24.9% in 2015 [37]. Following Iran's accession to the WHO's Tobacco Control Framework Convention (FCTC), various laws and strategies were enacted to control tobacco use. However, the long-term goals have not yet been achieved, and it seems that stricter rules and strategies, along with greater accuracy in their implementation, are needed [38].

The strength of the current study is that it included a large population-based cohort with repeated measurements of CVD risk factors over a substantial follow-up period. Furthermore, we used the Pooled Risk Equation recommended by ACC/AHA that has been validated in the TLGS before [16] and is widely used in clinical practice. However, there are some limitations. First, TLGS is comprised of a large representative sample of Tehranian residents, so generalizing the results to the total Iranian adult population, mainly rural individuals, should be done with caution. Second, we provided evidence on the trends of CVD risk score and risk factors among diabetic and non-diabetic subjects in an ongoing study that can increase the level of attention they pay to their health. Finally, in the current study, we did not investigate the cause-effect relationship between CVD risk factors and CVD risk, which is more difficult to understand through observational studies because of unmeasured confounding variables. However, since CVD risk is calculated using CVD risk factors in a prediction model directly, CVD risk depends on CVD risk factors mathematically, not causally. Therefore, our results are merely descriptive, and causality cannot be inferred.

In conclusion, this study shows that most risk factors improved in individuals with and without diabetes in the past two decades, and the results of these improvements can be seen in the favorable trends in the CVD risk score. In general, these improvements were more prominent in the diabetic population than in the non-diabetic population. However, the Iranian people have not yet reached their targets for control of CVD risk factors. More stringent lifestyle modifications and treatment strategies are needed to control cardiovascular risk factors, especially for primary preventions in the general population.

## Supplementary Information

**Additional file 1:** **Table S1.** Age-adjusted marginal means of the ACC/AHA CVD risk score and risk factors among diabetic and non-diabetic adults aged 40-79 separated by sex; Teheran Lipid and Glucose Study (1999 - 2018).

## Data Availability

The datasets used during the current study are available from the corresponding author on reasonable request.

## Abbreviations

CVD: Cardiovascular Diseases
ACC/AHA: The American College of Cardiovascular (ACC) and the American Heart Association (AHA)
NHLBI: The National Heart, Lung and Blood Institute
TLGS: Tehran Lipid and Glucose study
GEE: Generalized Estimating Equation Models
SBP: Systolic Blood Pressure
TC: Total Cholesterol
FPG: Fasting Plasma Glucose
WHO: The World Health Organization
FCTC: The WHO's Tobacco Control Framework Convention
