# Supplementary material for: The trend of 10-year cardiovascular risk among diabetic and non-diabetic participants in Tehran Lipid and glucose study: 1999–2018
Source: BMC Public Health. 2022 Mar 28;22:596. doi: 10.1186/s12889-022-12981-9 (PMC8961927; doi:10.1186/s12889-022-12981-9)
Supplement: Supplementary file 1 — Additional file 1: Table S1. Age-adjusted marginal means of the ACC/AHA CVD risk score and risk factors among diabetic and non-diabetic adults aged 40-79 separated by sex; Teheran Lipid and Glucose Study (1999 - 2018). [file 12889_2022_12981_MOESM1_ESM.docx]

**The Trend of 10-year Cardiovascular Risk among diabetic and non-diabetic participants in Tehran Lipid and Glucose Study: 1999-2018**

Fatemeh Koohi^1^, Karim Kohansal^2^, Marzieh Saei Ghare Naz^3^, Somayeh Derakhshan^4^, Fereidoun Azizi^5^, Davood Khalili^2*^

1. Obesity Research Center, Research Institute for Endocrine Sciences, Shahid Beheshti University of Medical Sciences, Tehran, Iran.
2. Prevention of Metabolic Disorders Research Center, Research Institute for Endocrine Sciences, Shahid Beheshti University of Medical Sciences, Tehran, Iran.
3. Reproductive Endocrinology Research Center, Research Institute for Endocrine Sciences, Shahid Beheshti University of Medical Sciences, Tehran, Iran.
4. Department of Epidemiology, School of Public Health and Safety, Shahid Beheshti University of Medical Sciences, Tehran, Iran.
5. Endocrine Research Center, Research Institute for Endocrine Sciences, Shahid Beheshti University of Medical Sciences, Tehran, Iran.

***Corresponding address:**

Davood Khalili, MD MPH PhD

Prevention of Metabolic Disorders Research Center,Research Institute for Endocrine Sciences, Shahid Beheshti University of Medical Sciences, Tehran, Iran.

Email address: [dkhalili@endocrine.ac.ir](mailto:dkhalili@endocrine.ac.ir)

**Additional file 1: Table S1**

**Table S1.** Age-adjusted marginal means of the ACC/AHA CVD risk score and risk factors among diabetic and non-diabetic adults aged 40-79 separated by sex; Teheran Lipid and Glucose Study (1999 - 2018)

|  | Sex | Diabetes status | Examination cycles | | | | | | P _trend_ | P _interaction_ |
| --- | --- | --- | --- | --- | --- | --- | --- | --- | --- | --- |
|  |  |  | **Ⅰ** | **Ⅱ** | **Ⅲ** | **Ⅳ** | **Ⅴ** | **Ⅵ** |  |  |
| ACC/AHA risk score | **Men** | Diabetics | 13.79 | 14.31 | 13.30 | 12.29 | 11.76 | 11.54 | <0.001 | 0.115 |
|  |  | Non-Diabetics | 6.85 | 6.80 | 6.53 | 5.95 | 5.74 | 5.01 | <0.001 |  |
|  | **Women** | Diabetics | 5.32 | 5.72 | 5.00 | 5.26 | 5.63 | 5.50 | <0.001 | <0.001 |
|  |  | Non-Diabetics | 3.20 | 2.94 | 2.65 | 2.52 | 2.30 | 1.61 | <0.001 |  |
| SBP | **Men** | Diabetics | 131.05 | 126.65 | 125.78 | 125.18 | 123.40 | 121.62 | <0.001 | 0.003 |
|  |  | Non-Diabetics | 124.87 | 122.34 | 121.76 | 121.40 | 121.79 | 119.80 | <0.001 |  |
|  | **Women** | Diabetics | 133.27 | 127.77 | 124.34 | 123.86 | 122.71 | 117.65 | <0.001 | 0<0.001 |
|  |  | Non-Diabetics | 126.82 | 121.85 | 118.62 | 119.85 | 119.64 | 114.40 | <0.001 |  |
| TC | **Men** | Diabetics | 219.61 | 201.57 | 197.24 | 195.49 | 191.21 | 182.63 | <0.001 | <0.001 |
|  |  | Non-Diabetics | 212.32 | 194.75 | 192.93 | 193.75 | 197.38 | 194.99 | <0.001 |  |
|  | **Women** | Diabetics | 241.94 | 218.84 | 212.79 | 202.78 | 193.32 | 184.00 | <0.001 | <0.001 |
|  |  | Non-Diabetics | 231.98 | 209.16 | 205.16 | 204.40 | 206.26 | 202.09 | <0.001 |  |
| HDL-C | **Men** | Diabetics | 37.69 | 34.64 | 37.02 | 41.94 | 43.13 | 41.82 | <0.001 | 0.375 |
|  |  | Non-Diabetics | 39.23 | 35.68 | 38.08 | 42.65 | 43.91 | 42.62 | <0.001 |  |
|  | **Women** | Diabetics | 45.28 | 41.06 | 43.46 | 48.74 | 50.75 | 49.41 | <0.001 | <0.001 |
|  |  | Non-Diabetics | 45.73 | 41.72 | 44.53 | 51.21 | 53.25 | 51.32 | <0.001 |  |
| FPG | **Men** | Diabetics | 152.53 | 159.77 | 150.41 | 156.01 | 149.11 | 148.42 | 0.067 | <0.001 |
|  |  | Non-Diabetics | 93.53 | 93.87 | 92.71 | 97.38 | 97.46 | 95.04 | <0.001 |  |
|  | **Women** | Diabetics | 164.17 | 156.54 | 152.03 | 150.61 | 143.30 | 134.20 | <0.001 | <0.001 |
|  |  | Non-Diabetics | 93.35 | 93.89 | 92.94 | 97.11 | 96.44 | 92.56 | <0.001 |  |
| Current Smoking, % | **Men** | Diabetics | 24 | 23 | 21 | 19 | 24 | 25 | 0.573 | 0.456 |
|  |  | Non-Diabetics | 22 | 24 | 23 | 21 | 22 | 24 | 0.890 |  |
|  | **Women** | Diabetics | 2 | 4 | 3 | 2 | 4 | 4 | 0.206 | 0.706 |
|  |  | Non-Diabetics | 3 | 3 | 3 | 3 | 3 | 3 | 0.643 |  |

SBP, Systolic Blood Pressure; TC, Total Cholesterol; HDL-C, High-Density Lipoprotein Cholesterol; FPG, Fasting Plasma Glucose. Models for assessment of time trend were fitted separately for diabetic and non-diabetic subjects, and age-adjusted marginal means and p-values for trend were reported. Meanwhile, the interaction of diabetes status with time was assessed by fitting a model including diabetes status, the follow-up time and their cross-product in a pooled model including both diabetic and non-diabetic participants, and p-values for the cross-product term were reported as p for trend.
